# Associations between maternal obesity and infectious morbidity in Zimbabwean infants

**DOI:** 10.1038/s41430-021-00907-4

**Published:** 2021-04-28

**Authors:** Thomas Althaus, Bernard Chasekwa, Ruairi C. Robertson, Robert Ntozini, Katie Greenland, Jean H. Humphrey, Andrew J. Prendergast

**Affiliations:** 1 London School of Hygiene and Tropical Medicine, London, UK; 2 Zvitambo Institute for Maternal and Child Health Research, Harare, Zimbabwe; 3 Blizard Institute, Queen Mary University of London, London, UK; 4 Department of International Health, Johns Hopkins Bloomberg School of Public Health, Baltimore, MD USA

**Keywords:** Risk factors, Infectious diseases, Obesity

## Abstract

The prevalence of overweight and obesity is increasing among reproductive-age women in sub-Saharan Africa. Whether maternal body mass index (BMI) influences the risk of infant infections in low- and middle-income countries (LMIC) is uncertain. We used data from a birth cohort of 5344 HIV-unexposed Zimbabwean infants with available data on maternal BMI, to calculate rates of sick clinic visits for infections during the first 12 months postpartum, and adjusted hazard ratios (aHR) for each maternal BMI group. Compared to infants of mothers with normal BMI, the rate of sick clinic visits for any infection progressively rose among infants of overweight (aHR 1.05; 95%CI 0.99, 1.11) and obese women (aHR 1.15; 95%CI 1.05, 1.25). Excess clinic attendances were particularly due to skin, respiratory and ear infections. Maternal obesity may therefore influence infant infectious morbidity in LMIC over the first year after birth.

## Introduction

The prevalence of overweight and obesity in low- and middle-income countries (LMIC) has markedly increased over recent decades [1]. In contrast to high-income countries, overweight and obesity in sub-Saharan Africa are more common in women compared to men [1]. Obesity in reproductive-age women is associated with an elevated risk of neonatal, perinatal, and infant death, in both high-income and LMIC settings [2, 3]. However, few studies have examined the influence of maternal overweight and obesity on infant infectious morbidity.

Excess adiposity is associated with chronic inflammation and immune dysfunction [4], which is evident in the placenta and umbilical cord blood of obese mothers [5]. This altered immune phenotype observed in maternal obesity may plausibly shape offspring immune development and subsequent infection risk [6]. Indeed, large studies from high-income countries have observed that each unit increase in body-mass index (BMI) of mothers during early pregnancy is associated with an 8.3% increased risk of early-onset sepsis in term infants [7]. Similarly, maternal obesity is associated with a higher risk of infant respiratory tract infections during the first year of life [8]. Longer-term follow-up studies have also shown that maternal overweight or obesity are independent risk factors for hospitalization for infections in children up to 5 years [9] and 18 years [10] of age.

Existing evidence is almost exclusively from high-income settings, while the burden of infectious diseases in children is far greater in LMIC. Here, we investigate associations between maternal overweight and obesity and infant infectious morbidity in a Zimbabwean birth cohort.

## Methods

This study used data from the ZVITAMBO trial [11]. Briefly, 14,110 mother–infant pairs were enrolled after delivery in Harare, Zimbabwe between 1997 and 2000. Mother–infant pairs were eligible if neither had acutely life-threatening conditions, the baby was a singleton with a birthweight >1500 g, and the mother planned to stay in Harare. Maternal written informed consent was obtained. The Medical Research Council of Zimbabwe, Institutional Review Board of Johns Hopkins Bloomberg School of Public Health, USA, and Montreal General Hospital, Canada, provided approvals. The trial was registered at clinicaltrials.gov (NCT00198718).

In this analysis, we included HIV-negative mothers with available anthropometry. Height was measured at delivery, and weight at 6-weeks postpartum. Maternal BMI was classified as underweight (<18.5 kg/m^2^); normal (18.5–24.99 kg/m^2^); overweight (25–29.99 kg/m^2^); or obese (≥30 kg/m^2^). At 6 weeks, then 3, 6, 9, and 12 months of age, mothers were asked whether their infant had attended a clinic, hospital, outpatient department, casualty, private doctor, or nurse since the previous study visit. The date and reason for each visit were determined from handheld records or, if these were not available, by maternal report. If a child was sick during a scheduled study visit, or if a sick child presented to the research clinic between scheduled visits, free treatment was provided and study staff recorded the reason for the visit. We were interested in all infant infections, which we classified as acute respiratory infection (ARI), tuberculosis, diarrhea, dysentery, ear infection, fever, lymphadenopathy, measles, other skin rashes, malaria, oral thrush, and “other” infection. Each reported visit for these illnesses contributed to the calculated rate of clinic visits for “any infection”. Rates of attendance for specific infections were calculated for the most common causes: ARI, diarrhea (including dysentery), ear infections, skin rash (including measles), and fever; other categories had too few events to calculate infection-specific visits rates.

The number of clinic visits was compared between maternal BMI groups, and rates per 1000 child-months calculated. An exponential regression model with gamma frailty was used to obtain unadjusted and adjusted hazard ratios (aHR) for each group, using normal BMI as the reference group. We considered all variables associated with maternal BMI and the outcome of interest as potential confounders. We tested each variable individually and included those affecting the point estimates of the outcomes or their 95% confidence intervals. Covariates selected for the final models were gestational age, birth weight, parity, maternal education, and household income. Analysis was undertaken using STATA version 12 (College Station, Texas, USA).

## Results

Among 14,110 women, 9208 (65.3%) were HIV-negative; of these, 5344 (58.0%) had BMI data available and were included in this analysis. Supplementary Table 1 shows the differences between women with and without BMI data. Overall, 240 (4.5%) women were underweight; 3552 (66.5%) had normal BMI; 1171 (21.9%) were overweight and 381 (7.1%) were obese. Table 1 shows the baseline characteristics of mothers and infants.

**Table 1:** Baseline characteristics of infants and mothers.

	Maternal BMI				*P*-value^1^
	Underweight (*n* = 240)	Normal (*n* = 3552)	Overweight (*n* = 1171)	Obese (*n* = 381)	
*Infant characteristics*					
Male sex, *n* (%)	129 (53.7)	1844 (51.9)	602 (51.4)	195 (51.3)	0.900
Gestational age, weeks, mean (SD)	39.0 (1.5)	39.3 (1.4)	39.4 (1.3)	39.6 (1.4)	0.060
Preterm (<37 weeks), *n* (%)	30 (12.5)	220 (6.2)^a^	63 (5.4)^a^	18 (4.7)^a^	0.006
Apgar score, median (IQR)	10 (9–10)	10 (9–10)	10 (9–10)	10 (9–10)	0.250
Feeding pattern, *n* (%):^2^					0.447
– Exclusive breastfeeding	13 (5.4)	186 (5.2)	63 (5.4)	31 (8.1)	
– Predominant breastfeeding	44 (18.3)	768 (21.6)	261 (22.3)	81 (21.3)	
– Mixed breastfeeding	128 (53.3)	1,892 (53.3)	636 (54.3)	205 (53.8)	
Birth length, cm, mean (SD)	47.9 (2.4)	48.5 (2.6)	48.9 (2.5)^a^	49.2 (2.5)^a^	<0.001
Birth weight, grams, mean (SD)	2819 (431)	2979 (435)^a^	3109 (440)^a,b^	3181 (490)^a,b^	0.010
Infant Vitamin A, *n* (%)^3^	130 (54.2)	1725 (48.6)	608 (51.9)	194 (50.9)	0.100
*Maternal characteristics*					
Age, years, median (IQR)	21.5 (19.2–24.4)	22.5 (20.0–25.9)^a^	24.9 (21.4-29.6)^a,b^	28.5 (24.1–34.3)^a,b,c^	<0.001
Parity, median (IQR)	1 (1–2)	1 (1–2)^a^	2 (1–3)^a,b^	3 (2–4)^a,b,c^	<0.001
Weight, kg, mean (SD)^4^	46.8 (5.2)	56.4 (6.2)^a^	68.5 (5.9)^a,b^	84.7 (9.2)^a,b,c^	<0.001
Height, cm, mean (SD)	162.7 (8.9)	160.2 (6.4)^a^	159.4 (5.9)^a,b^	159.7 (6.1)^a^	<0.001
MUAC, cm, mean (SD)	23.1 (2.0)	25.1 (2.2)^a^	28.2 (2.3)^a,b^	29.4 (4.4)^a,b,c^	<0.001
Married or stable union, *n* (%)	222 (92.5)	3351 (94.3)	1107 (94.5)	369 (96.9)^a,b^	0.005
Education: secondary, *n* (%)	205 (85.4)	3019 (85.0)	964 (82.3)	271 (71.1)^a,b,c^	<0.001
Employed, *n* (%)	21 (8.8)	319 (9.0)	154 (13.2)^a,b^	70 (18.4)^a,b^	<0.001
Household income/month, US$, median (IQR)	963 (650–1612)	1056 (700–1738)	1186 (780–1974)^a,b^	1468 (949–2426)^a,b,c^	<0.001
Husband secondary education, *n* (%)	229 (95.4)	3294 (92.7)	1076 (91.9)	312 (81.9)^a,b,c^	<0.001
Husband employment, *n* (%)	150 (62.5)	2068 (58.2)	715 (61.1)	242 (63.5)	0.29

^1^P values were calculated using the chi-squared test for binary variables, ANOVA for comparison of means, and Kruskall–Wallis tests for comparison of medians. A post-hoc Bonferroni test was conducted if overall *p*-value <0.05; the superscript letter denotes a significant difference between that group and ^a^underweight, ^b^normal weight, ^c^overweight, and ^d^obese.

^2^Detailed feeding information was collected from mothers at 6 weeks, 3 months, and 6 months of age, including whether any of 22 liquids (water, juice, tea, cooking oil), milk (formula, fresh, tinned), medicines (traditional, oral rehydration solution, prescribed) or solid foods (porridge, sadza, fruit, vegetables, meat, eggs) had been given to the infant. Breastfeeding was defined as exclusive, predominant, or mixed at 3 months of age. Data were not available at 3 months of age for 23% underweight, 15% normal BMI, 18% overweight, and 17% obese women.

^3^The randomization in the original ZVITAMBO trial, to Aa, Pa, Ap, and Pp (where A is maternal Vitamin A, P is maternal placebo, a is infant Vitamin A and p is infant placebo).

^4^Maternal weight was measured 6 weeks postpartum.

Table 2 shows the rates of clinic attendances during infancy and aHRs for each maternal BMI category. The rate of clinic visits for any infection progressively rose with increasing BMI categories. In adjusted analyses, clinic attendance for any infection tended to be higher among infants of overweight women (aHR 1.05; 95%CI 0.99, 1.11) and was significantly higher among infants of obese women (aHR 1.15; 95%CI 1.05, 1.25). There was no evidence of increased clinic attendance among infants of underweight mothers.

**Table 2:** Clinic visits for infections during the first year after birth by maternal BMI category.

Type of infection	Number of visits^a^	Rate/1000 child-months	Crude hazard ratio	Adjusted hazard ratio^b^
*Any infection*^c^				
Underweight	485	168.4	0.98 (0.88–1.08)	1.01 (0.90–1.14)
Normal	7323	172.3	1	1
Overweight	2499	178.2	1.03 (0.98–1.09)	1.05 (0.99–1.11)
Obese	878	193.8	1.13 (1.04–1.22)	1.15 (1.05–1.25)
*Diarrhea*^d^				
Underweight	70	24.3	1.19 (0.91–1.55)	1.30 (0.95–1.76)
Normal	874	20.5	1	1
Overweight	265	18.9	0.92 (0.79–1.07)	0.96 (0.81–1.13)
Obese	96	21.1	1.03 (0.82–1.29)	1.12 (0.86–1.46)
*ARI*				
Underweight	288	100	0.90 (0.79–1.03)	0.96 (0.82–1.11)
Normal	4706	110.6	1	1
Overweight	1539	109.7	0.99 (0.93–1.06)	0.99 (0.92–1.06)
Obese	553	121.4	1.10 (1.00–1.21)	1.09 (0.98–1.22)
*Fever*				
Underweight	43	14.9	1.06 (0.76–1.47)	0.86 (0.56–1.32)
Normal	602	14.2	1	1
Overweight	195	13.9	0.98 (0.83–1.17)	1.00 (0.82–1.21)
Obese	78	17.1	1.21 (0.94–1.56)	1.10 (0.82–1.48)
*Ear infection*				
Underweight	15	5.2	0.71 (0.39–1.28)	0.93 (0.48–1.83)
Normal	314	7.4	1	1
Overweight	134	9.5	1.30 (1.01–1.66)	1.41 (1.05–1.89)
Obese	45	9.9	1.34 (0.90–1.99)	1.20 (0.74–1.93)
*Skin infection*^e^				
Underweight	80	27.8	1.00 (0.76–1.29)	0.90 (0.65–1.24)
Normal	1196	28.1	1	1
Overweight	432	30.8	1.10 (0.96–1.25)	1.09 (0.94–1.27)
Obese	169	37.2	1.32 (1.09–1.61)	1.46 (1.17–1.82)

*ARI* Acute respiratory infection.

^a^Number of visits between birth and 12 months of age are calculated among 240 children born to underweight mothers; 3552 born to mothers in normal BMI range; 1171 born to overweight mothers, and 381 born to obese mothers.

^b^Adjusted hazard ratios were calculated using gestational age, birth weight, maternal education, income, and parity as covariates.

^c^Acute respiratory infection, tuberculosis, acute and persistent diarrhea, dysentery, ear infection, fever, lymphadenopathy, measles, other skin rashes, malaria, oral thrush, and other infection.

^d^Including dysentery.

^e^Measles and other skin rashes.

Infants born to obese compared to normal-weight mothers had a significantly higher rate of clinic attendance for skin rash (aHR 1.46 (95%CI 1.17, 1.82)), and some evidence of increased clinic attendance for ARI (aHR 1.09; 95%CI 0.98, 1.22). Infants of overweight compared to normal-weight mothers had a significantly higher rate of clinic attendance for ear infections (aHR 1.30; 95%CI 1.01, 1.66).

## Discussion

Here, we report that maternal BMI is associated with infant infectious morbidity in the first year of life in a Zimbabwean birth cohort. Specifically, infants of overweight and obese mothers had more clinic attendances for infections compared to infants of mothers with normal BMI, particularly for skin, ear, and respiratory infections.

Maternal overweight and obesity have previously been associated with stillbirth, fetal death, and preterm birth, in studies conducted predominantly in high-income countries [2, 12]. In the neonatal period, the risk of early-onset sepsis and mortality are also elevated in the offspring of overweight and obese mothers [2, 7, 13]. In an analysis of data from 27 sub-Saharan African countries, maternal obesity was associated with increased odds of neonatal death (adjusted odds ratio 1.46, 95%CI 1.11–1.91), with infections likely to be one of the underlying causes [3]. The longer-term influence of maternal BMI on health in childhood is more difficult to delineate due to the influence of other confounding factors [6]. However, previous studies from high-income settings have reported more hospitalizations for infectious diseases in children born to obese mothers during follow-up through 18 years of age [9, 10, 14]. Maternal-reported wheeze, prolonged cough, and lower respiratory tract infections (but not croup, ear infections, diarrhea, or vomiting) among infants were associated with higher maternal BMI in a UK study [8].

Data on infectious morbidity from LMIC are lacking, despite the high burden of disease. In 2016, lower respiratory tract infections caused over 650,000 deaths in children under-five worldwide [15], and diarrhea was the fifth leading cause of death [16]. During the ongoing “nutrition transition”, when maternal overweight and obesity are increasing in LMIC, it is critical to identify early-life risk factors for childhood infection. We believe this is the first study reporting an association between maternal BMI and infant infections in LMIC.

Despite observational data supporting an association between maternal BMI and childhood infection in high-income settings, the underlying mechanisms are poorly understood. Obesity prior to and during pregnancy is associated with maternal immune deficits including reduced natural-killer cells and CD8 + T-cells [17, 18]. Consequently, infants born to obese mothers have fewer eosinophils and CD4 T-cells, and impaired responses to stimulation from toll-like receptor ligands, suggesting that maternal BMI influences neonatal immune ontogeny [5, 19]. Neonates of overweight and obese mothers also display increased “inflammatory scores” characterized by elevated pro-inflammatory and reduced anti-inflammatory biomarkers [20]. This altered immune landscape in infants of obese mothers may influence early-life infection susceptibility. Obesity also increases the risk of maternal infections [21, 22]. This may enable vertical transmission of pathogens to the offspring and thereby increase the risk of infant infection [23]. An alternative explanation is that overweight and obese mothers had different health-seeking behaviors. Mothers with greater BMI had higher household income in our study, which is associated with better access to healthcare [24]; however, the elevated risk of infections remained after adjusting for income and education.

Our results are strengthened by the large cohort and regular postnatal follow-up. In addition, free clinical care during the trial and review of medical records from clinic attendances allowed the collection of robust data on infant illnesses. However, there are also limitations. First, data on maternal BMI were missing for 41.7% of eligible mothers from the trial population, who differed in several respects. Excluded women were younger, with lower parity, mid-upper arm circumference, and infant birth weight, and differences in breastfeeding; all these factors could influence infant infectious morbidity. Second, BMI was measured 6-weeks postpartum, which may not reflect pre-pregnancy weight or gestational weight gain. Third, clinic visits for specific symptoms such as diarrhea and skin rash were assumed to indicate infections, but some episodes may have been due to non-infectious causes. Finally, although we adjusted our models for gestational age, birth weight, parity, maternal education, and household income, there may still be unmeasured confounding which explains the associations reported.

In summary, we report that overweight and obesity in Zimbabwean women are associated with more clinic attendances for infant infections during the first year after birth. Whether these findings are predominantly driven by altered infant immune development, increased pathogen transmission, or differences in health-seeking behavior requires further investigation. Regardless of the underlying causes, our results highlight the potential impact of maternal obesity on the health of infants beyond the newborn period. Given the increasing prevalence of obesity in LMIC, especially among reproductive-age women, there may be increased pressure on resource-limited health systems due to effects on infant health. Further studies evaluating the influence of maternal overweight and obesity on infant morbidity, mortality, growth, and development in LMIC are required.

## Supplementary information

Supplementary Table 1
