# Supplementary material for: Associations between maternal obesity and infectious morbidity in Zimbabwean infants
Source: Eur J Clin Nutr. 2021 Apr 28;76(2):328–33. doi: 10.1038/s41430-021-00907-4 (PMC8821001; doi:10.1038/s41430-021-00907-4)
Supplement: Supplementary file 1 — Supplementary Table 1 [file 41430_2021_907_MOESM1_ESM.docx]

**Supplementary Table 1: Baseline characteristics of infants and mothers with and without available maternal BMI data**

|  | **No BMI available** | **BMI available** | **P-value** |
| --- | --- | --- | --- |
| **(n=3865)** | **(n=5344)** |  |  |
| **Infant characteristics** |  |  |  |
| Male sex, n (%) | 2004 (51.9) | 2770 (51.9) | 0.982 |
| Gestational age,  weeks, mean (SD) | 39.3 (1.4) | 39.3 (1.4) | 0.108 |
| Preterm (<37 weeks), n (%) | 248 (6.5) | 331 (6.3) | 0.647 |
| Apgar score, median (IQR) | 10 (9-10) | 10 (9-10) | 0.556 |
| Feeding pattern, n (%) |  |  | <0.001 |
| - Exclusive breastfeeding | 79 (2.0) | 293 (5.5) |  |
| - Predominant breastfeeding | 278 (7.2) | 1154 (21.6) |  |
| - Mixed breastfeeding | 925 (23.9) | 2861 (53.5) |  |
| Birth length, cm, mean (SD) | 48.6 (2.6) | 48.6 (2.6) | 0.578 |
| Birth weight, grams,  mean (SD) | 2980 (446) | 3015 (447) | <0.001 |
| Infant Vitamin A, n (%) | 1943 (50.3) | 2657 (49.7) | 0.601 |
| **Maternal characteristics** |  |  |  |
| Age, years, mean (SD) | 23.6 (5.1) | 24.5 (5.5) | <0.001 |
| Parity, median (IQR) | 1 (1-2) | 2 (1-3) | <0.001 |
| MUAC, cm, mean (SD) | 25.9 (3.0) | 26.1 (3.1) | 0.001 |
| Married or stable union, n (%) | 3630 (94.3) | 5049 (94.8) | 0.338 |
| Education: Secondary, n (%) | 3157 (81.8) | 4455 (83.5) | 0.033 |
| Employed, n (%) | 630 (16.4) | 960 (18.0) | 0.040 |
| Household income/month,  US$, median (IQR) | 1097 (695-1936) | 1115 (731-1845) | 0.939 |
| Husband secondary education, n (%) | 3529 (93.4) | 4860 (92.9) | 0.330 |
| Husband employment, n (%) | 3552 (93.4) | 4901 (93.1) | 0.580 |
